# Expression and clinical association of programmed cell death-1, programmed death-ligand-1 and CD8^+^ lymphocytes in primary sarcomas is subtype dependent

**DOI:** 10.18632/oncotarget.19071

**Published:** 2017-07-07

**Authors:** Anke E.M. van Erp, Yvonne M.H. Versleijen-Jonkers, Melissa H.S. Hillebrandt-Roeffen, Laurens van Houdt, Mark A.J. Gorris, Laura S. van Dam, Thomas Mentzel, Marije E. Weidema, C. Dilara Savci-Heijink, Ingrid M.E. Desar, Hans H.M. Merks, Max M. van Noesel, Janet Shipley, Winette T.A. van der Graaf, Uta E. Flucke, Friederike A.G. Meyer-Wentrup

**Affiliations:** ^1^ Department of Medical Oncology, Radboud University Medical Center, Nijmegen, The Netherlands; ^2^ Department of Tumor Immunology, Radboud Institute of Molecular Life Sciences, Radboud University Medical Center, Nijmegen, The Netherlands; ^3^ Princess Máxima Center for Pediatric Oncology, Utrecht, The Netherlands; ^4^ Dermatopathology Bodensee, Friedrichshafen, Germany; ^5^ Department of Pathology, Academic Medical Center, Amsterdam, The Netherlands; ^6^ Department of Pediatric Oncology, Emma Children’s Hospital-Academic Medical Center, University of Amsterdam, Amsterdam, The Netherlands; ^7^ Sarcoma Molecular Pathology Team, Divisions of Molecular Pathology and Cancer Therapeutics, Institute of Cancer Research, London, United Kingdom; ^8^ The Institute of Cancer Research and The Royal Marsden NHS Foundation Trust, London, United Kingdom; ^9^ Department of Pathology, Radboud University Medical Center, Nijmegen, The Netherlands

**Keywords:** sarcoma, desmoplastic small round cell tumor (DSRCT), programmed cell death-1 (PD-1), programmed death ligand-1 (PD-L1), immune checkpoint blockade

## Abstract

In order to explore the potential of immune checkpoint blockade in sarcoma, we investigated expression and clinical relevance of programmed cell death-1 (PD-1), programmed death ligand-1 (PD-L1) and CD8 in tumors of 208 sarcoma patients. Primary untreated osteosarcoma (*n* = 46), Ewing sarcoma (*n* = 32), alveolar rhabdomyosarcoma (*n* = 20), embryonal rhabdomyosarcoma (*n* = 77), synovial sarcoma (*n* = 22) and desmoplastic small round cell tumors (DSRCT) (*n* = 11) were examined immunohistochemically. PD-L1 expression was predominantly detected in alveolar and embryonal rhabdomyosarcomas (15% and 16%, respectively). In the alveolar subtype PD-L1 expression was associated with better overall, event-free and metastases-free survival. PD-1 expression on lymphocytes was predominantly seen in synovial sarcomas (18%). High levels of CD8+ lymphocytes were predominantly detected in osteosarcomas (35%) and associated with worse event-free survival in synovial sarcomas. Ewing sarcoma and DSRCTs showed PD-1 on tumor cells instead of on tumor infiltrating lymphocytes. Overall, expression and clinical associations were found to be subtype dependent. For the first time PD-1 expression on Ewing sarcoma (19%) and DSRCT (82%) tumor cells was described.

## INTRODUCTION

Immune checkpoint inhibitors have become major players in cancer treatment with FDA-approval of nivolumab for the treatment of recurrent Hodgkin’s lymphoma, non-small cell lung cancer (NSCLC), metastatic renal cell carcinoma, and melanoma and recent approval of pembrolizumab for the treatment of NSCLC and melanoma. Tumor cells can generate an immune suppressive environment by upregulation of the programmed death ligand-1 (PD-L1) on their cell membrane. Interaction with its receptor programmed cell death-1 (PD-1) on CD8^+^ T lymphocytes (TL) renders the TL inactive, thus preventing killing of tumor cells [1]. Inhibition of this interaction by anti-PD-1 antibodies can reinstate cytotoxic activity of TLs leading to subsequent killing of tumor cells [2, 3].

Sarcomas are rare, highly malignant bone and soft tissue tumors with a relatively high incidence in children, adolescents and young adults (AYA). Over 70 subtypes exist. The most common primary bone tumors in children and adolescents are osteosarcoma and Ewing sarcoma with a 5-year survival of 60–78% for localized disease and 20-30% for metastatic disease [4, 5]. Rhabdomyosarcoma is the most common soft tissue sarcoma (STS) in pediatric patients with approximately 80% presenting with embryonal rhabdomyosarcoma and 20% with alveolar rhabdomyosarcoma. Low and intermediate risk groups show a 5- and 4-year event-free survival of 88% and 76%, respectively, while the high risk group shows a 5-year survival of 25–40% [6, 7]. The most common non-rhabdomyosarcoma STS is the synovial sarcoma with a peak incidence in adolescents and young adults. Similar to rhabdomyosarcoma, survival rates depend on low risk or high risk status with 88% and 18% 5-year disease-free survival, respectively [8]. Desmoplastic small round cell tumors (DSRCTs) are a very rare subtype with an incidence peak in young adult patients and a male predominance. Survival rates are limited to a 5-year survival of 15%, limited treatment options exist and tumors respond poorly to chemotherapy [9, 10]. Genetically several sarcoma subtypes are characterized by specific gene fusions. Ewing sarcoma is most commonly characterized by the *EWSR1-FLI1* or *EWSR1-ERG* translocation [11]. Fusion-positive alveolar rhabdomyosarcoma is characterized by the *PAX3-FOXO1* or *PAX7-FOXO1* translocation and synovial sarcomas by the *SS18-SSX1* or *SS18-SSX2* translocation [8, 12]. Similar to Ewing sarcoma, DSRCTs present with an *EWSR1* translocation that in DSRCTs fuses with the *WT1* gene [9]. Multimodality treatment is the standard therapy in sarcoma consisting of (neo)adjuvant chemotherapy, surgery and/or radiotherapy. All chemotherapy regimens consist of multiple chemotherapeutic agents specified for each individual sarcoma subtype. Despite the use of multimodality treatment the survival rates have stagnated and the need for new therapeutic options is high.

Little is known about clinical effects of PD-1 blockade and expression of PD-1, PD-L1 and CD8^+^ lymphocytes in tumors of young sarcoma patients. Recent studies describing their expression in sarcomas show conflicting data on expression levels and clinical associations. Paydas *et al.* described PD-1 and PD-L1 expression in a variety of sarcoma subtypes with an overall expression of PD-1 in 17% and PD-L1 in 29% of the tumors (*n =* 65). Expression per subtype ranged from 20–66%, 25–33% and 20–33% for PD-1, PD-L1 and PD-1^+^PD-L1^+^, respectively, and no correlation with clinical outcome was observed [13]. In contrast, Kim *et al.* described PD-L1 in a larger group of STS (*n =* 82) with expression ranging from 33–53% and an association with worse overall survival (OS) [14]. Previous to these studies, the presence of both PD-L1 expression and CD8^+^ lymphocytes was described in a variety of sarcomas (*n =* 59), with relatively high PD-L1 expression for osteosarcoma, Ewing sarcoma and rhabdomyosarcoma. PD-L1 expression per sarcoma subtype ranged from 47–89% and was not associated with clinical outcome. However, PD-L1 in combination with high CD8 levels were associated with better survival which further increased if more than 10% of the CD8^+^ lymphocytes were PD-1^+^ [15]. Others examined either only PD-L1 or both PD-1 and PD-L1 in multiple tumor or sarcoma subtypes. Expression ranged from 0–17% for PD-L1 or 0–75% for PD-1 and 0–100% for PD-L1 [16, 17]. The latter two studies included osteosarcoma, Ewing sarcoma, rhabdomyosarcoma, synovial sarcoma and DSRCT, although limited numbers were included in the study of Movva *et al.* [16]. No clinical associations were described, and for DSRCTs only PD-L1 expression was determined.

Taken together, the above-mentioned studies do suggest a potential therapeutic role for PD-1 inhibitors in (a subgroup of) sarcomas. However, differences in expression levels and clinical association were found and are possibly due to variations in sample size, the primary antibody used, or access to patient data (Supplementary Table 1). In order to further explore and clarify the potential of PD-1 blockade in young sarcoma patients, the expression and clinical association of PD-1, PD-L1 and the presence of CD8^+^ lymphocytes were assessed in (high-grade) primary untreated tumors (*n =* 208), consisting of osteosarcoma (*n =* 46), Ewing sarcoma (*n =* 32), alveolar rhabdomyosarcoma (*n =* 20), embryonal rhabdomyosarcoma (*n =* 77) and synovial sarcoma (*n =* 22). In addition, we are the first to describe the expression of PD1 and CD8 in a cohort of DSRCTs (*n =* 11).

## RESULTS

### PD-1 and PD-L1 expression and the presence of CD8^+^ lymphocytes in primary sarcoma

**Figure 1:**
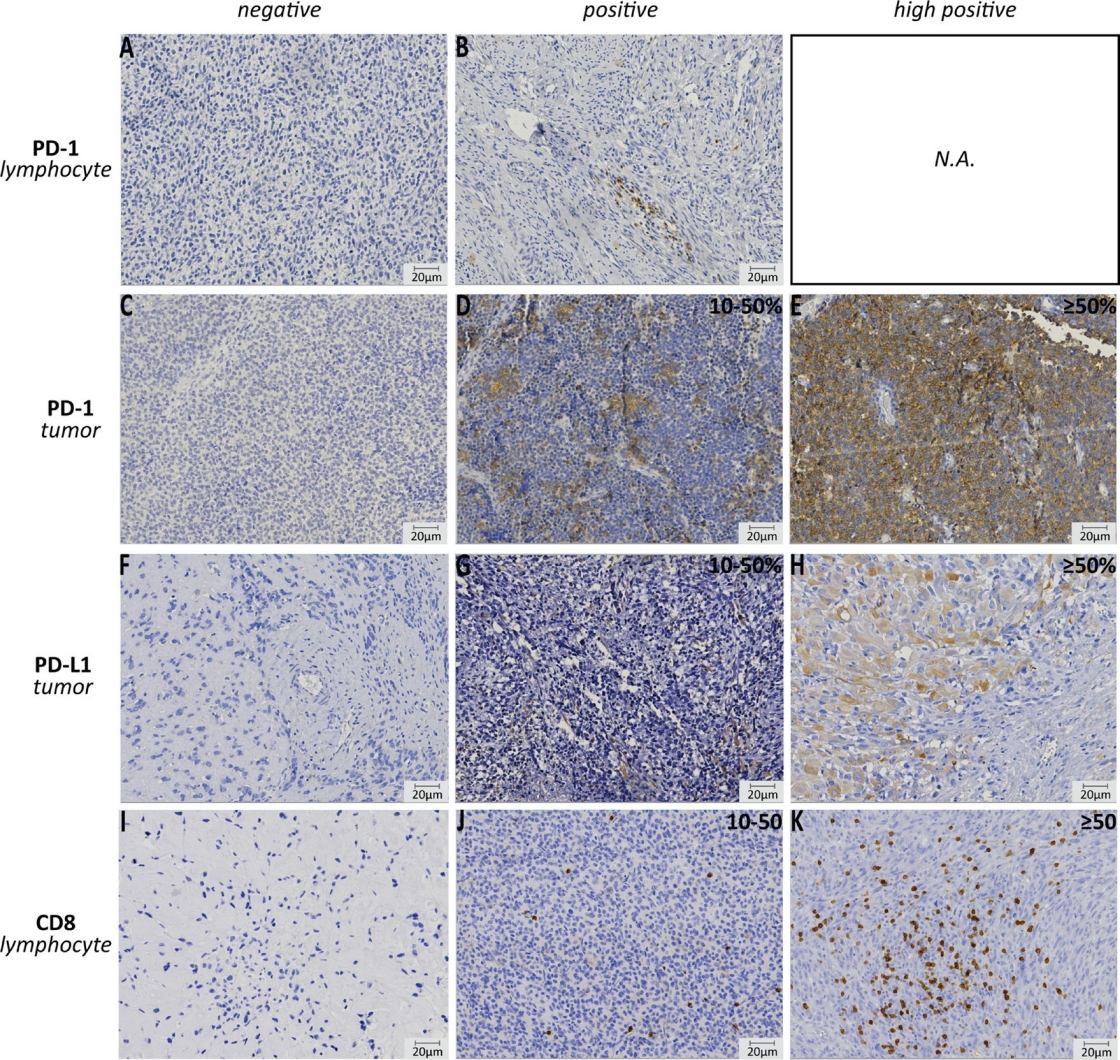
Immunohistochemistry of PD-1, PD-L1 and CD8 expression Examples of negative and positive PD-1 expression on lymphocytes (embryonal rhabdomyosarcoma, **A**–**B**); negative, positive (10–50%) and high (≥ 50%) PD-1 expression on tumor cell (Ewing sarcoma, **C**–**E**); negative, positive (10–50%) and high (≥ 50%) PD-L1 expression on tumor cell (embryonal rhabdomyosarcoma, **F**–**H**); negative, positive (10–50) and high (≥ 50) number of CD8^+^ lymphocytes in the tumor (osteosarcoma, **I**–**K**). N.A.: not applicable.

**Figure 2:**
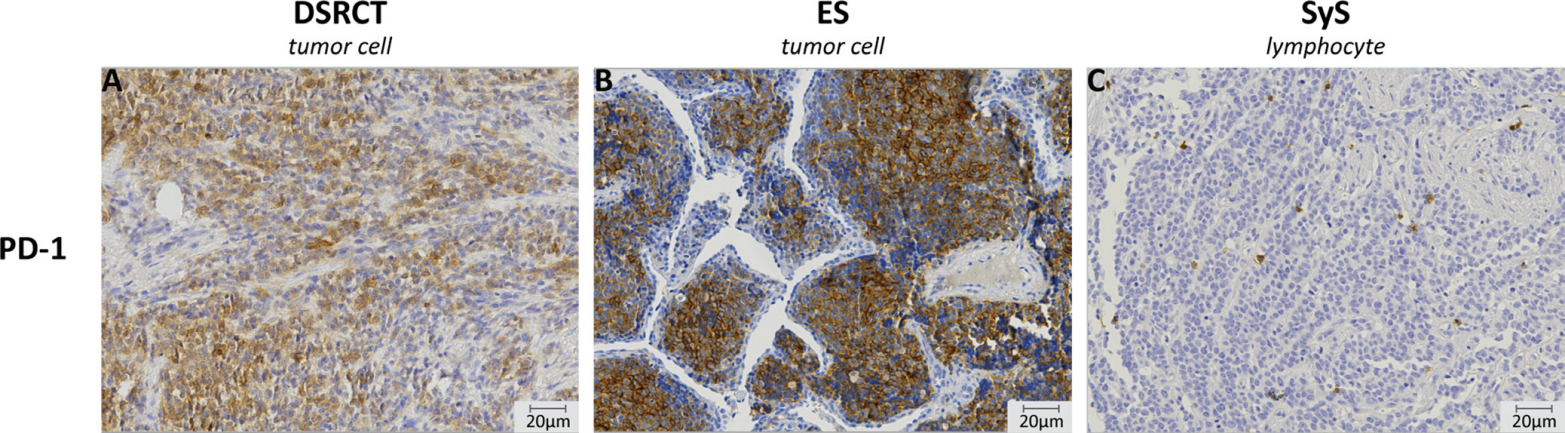
Immunohistochemistry of PD-1 expression on the tumor cell and on tumor infiltrating cells PD-1 expression was observed to be present on the tumor cells in both desmoplastic small round cell tumor (DSRCT) (**A**) and Ewing sarcoma (ES) (**B**). All other subtypes showed PD-1 expression on tumor infiltrating cells. An image of PD1^+^ lymphocytes in synovial sarcoma (SyS) is given (**C**). Images were taken at 20× magnification.

PD-1 and PD-L1 expression and the presence of CD8^+^ lymphocytes were assessed in primary osteosarcoma (*n =* 46), Ewing sarcoma (*n =* 32), alveolar rhabdomyosarcoma (*n =* 20), embryonal rhabdomyosarcoma (*n =* 77), synovial sarcoma (*n =* 22) and DSRCT (*n =* 11) and subdivided in negative (0–10 (%)), positive (10–50 (%)) or high positive (≥ 50 (%)) expression (Table 1 and Figure 1). Individual subtypes showed varying levels of expression, with the most prominent expression in DSRCTs (Table 2). Interestingly, nine out of 11 DSRCTs expressed PD-1 on the tumor in 10–50% of the tumor (9%) or in ≥ 50% of the tumor (PD-1_high_) (73%) (Figure 2A). One DSRCT showed both PD-1 and PD-L1 expression on the tumor and CD8^+^ lymphocytes in the tumor. Overall, seven DSRCTs had CD8^+^ lymphocytes in the tumor, with one expressing high levels of CD8 (CD8_high_). PD1 expression on the tumor was also detected in 19% of Ewing sarcomas (6% positive and 13% high positive) (Figure 2B). PD-1 expression on tumor infiltrating lymphocytes (TILs) was predominantly detected in synovial sarcomas (18%) (Figure 2C). Sixteen percent of embryonal rhabdomyosarcomas expressed PD-L1, with high PD-L1 expression (PD-L1_high_) in 6% of all examined cases. All subtypes showed CD8^+^ lymphocytes in the tumor in at least 30% of cases. In addition, a subset of each subtype expressed CD8_high_, with the most prominent expression in osteosarcoma (35%) (Table 2).

**Table 1:** Patient characteristics per individual sarcoma subtype

Tumor type	Characteristics		*N* (%)
**OST (*N* = 46)**	Gender	Male	24 (52)
		Female	22 (48)
	Age at diagnosis	< 18 y	14 (30)
		≥ 18 y	32 (70)
	Metastases	Yes	24 (52)
		No	22 (48)
	Initial metastases		3 (13)^a^
	Follow-up data available	OS	46 (100)
		EFS	46 (100)
**ES (*N* = 32)**	Gender	Male	17 (53)
		Female	15 (47)
	Age at diagnosis	< 18 y	25 (78)
		≥ 18 y	7 (22)
	Translocation	EWSR1-FLI1	30 (94)
		EWSR1-ERG	1 (3)
		Positive (not specified)	1 (3)
	Metastases	Yes	11 (34)
	Initial metastases	No	21 (66) 5 (45)^a^
	Follow-up data available	OS	32 (100)
		EFS	32 (100)
**ARMS (*N* = 20)**	Gender	Male	10 (50)
		Female	10 (50)
	Age at diagnosis	< 18 y	13 (65)
		≥ 18 y	7 (35)
	Translocation	PAX3-FOXO1	17 (85)
		PAX7-FOXO1	1 (5)
		Positive (not specified)	2 (10)
	Metastases	Yes	9 (45)
		No	5 (25)
		Unknown	6 (30)
	Initial metastases		4 (44)^a^
	Follow-up data available	OS	20 (100)
		EFS	17 (85)
**ERMS (*N* = 77)**	Gender	Male	51 (66)
		Female	26 (34)
	Age at diagnosis	< 18 y	63 (82)
		≥ 18 y	14 (18)
	Metastases	Yes	4 (5)
		No	46 (60)
		Unknown	27 (35)
	Initial metastases		2 (50)^a^
	Follow-up data available	OS	50 (64)
		EFS	50 (64)
**SyS (*N* = 22)**	Gender	Male	14 (64)
		Female	8 (36)
	Age at diagnosis	< 18 years	7 (32)
		≥ 18 years	15 (68)
	Translocation	SS18-SSX1	17 (77)
		SS18-SSX2	5 (23)
	Metastases	Yes	10 (45)
		No	11 (50)
		Unknown	1 (5)
	Initial metastases		0 (0)^a^
	Follow-up data available	OS	21 (95)
		EFS	22 (100)
**DSRCT (*N* = 11)**	Gender	Male	7 (64)
		Female	4 (36)
	Age at diagnosis	< 18 y	4 (36)
		≥ 18 y	7 (64)
	Translocation	EWSR1-WT1	11 (100)
	Metastases	Yes	6 (55)
		No	1 (9)
		Unknown	4 (36)
	Initial metastases		6 (55)^a^
	Follow-up data available	OS	8 (73)
		EFS	8 (73)

N: number of primary tumors, OST: osteosarcoma, ES: Ewing sarcoma, ARMS: alveolar rhabdomyosarcoma, ERMS: embryonal rhabdomyosarcoma, SyS: synovial sarcoma, DSRCT: desmoplastic small round cell tumor, DOD: death of disease, CR: complete response, PD: progressive disease, ^a^percentage calculation: (total with initial metastases/total with metastases)*100%.

**Table 2:** PD-1, PD-L1 expression and the presence of CD8^+^ lymphocytes in primary sarcoma tumors

Subtype	*N*	PD-1 lymphocytes (%)	PD-1 tumor^a^ (%)	PD-1_high_ tumor^b^ (%)	PD-L1 tumor^a^ (%)	PD-L1_high_ tumor^b^ (%)	CD8 (10–50)^c^ (%)	CD8_high_ (≥ 50)^c^ (%)	PD-1^+^PD-L1^+^ (%)	PD-1^+^CD8^+^ (%)	PD-L1^+^CD8^+^ (%)	PD-1^+^PD-L1^+^CD8^+^ (%)
**OST**	46	2 (4)	0 (0)	0 (0)	4 (9)	0 (0)	15 (33)	16 (35)	1 (2)	2 (4)	4 (9)	1 (2)
**ES**	32	0 (0)	2 (6)	4 (13)	0 (0)	0 (0)	12 (38)	1 (3)	0 (0)	4 (12)	0 (0)	0 (0)
**ARMS**	20	1 (6)	0 (0)	0 (0)	4 (15)	0 (0)	6 (30)	1 (5)	1 (5)	1 (5)	2 (10)	1 (5)
**ERMS**	77	5 (6)	0 (0)	0 (0)	8 (10)	5 (6)	23 (30)	6 (8)	2 (3)	5 (6)	8 (10)	2 (3)
**SyS**	22	4 (18)	0 (0)	0 (0)	0 (0)	0 (0)	9 (41)	1 (5)	0 (0)	3 (14)	0 (0)	0 (0)
**DSRCT**	11	0 (0)	1 (9)	8 (73)	2 (18)	0 (0)	6 (55)	1 (9)	1 (9)	6 (55)	1 (9)	1 (9)

*N* = total number of primary tumors, OST: osteosarcoma, ES: Ewing sarcoma, ARMS: alveolar rhabdomyosarcoma, ERMS: embryonal rhabdomyosarcoma, SyS: synovial sarcoma, ^a^10–50% positive tumor cells, ^b^ ≥ 50% positive tumor cells, ^c^in the tumor.

### Correlation between PD-1, PD-L1 and CD8 expression

Embryonal rhabdomyosarcoma showed a significant correlation between PD-1 on TILs (PD-1_TIL_) and CD8^+^ lymphocytes in the tumor (*p =* 0.006) and a trend for PD-L1 and CD8^+^ (*p =* 0.065). Other subtypes showed no correlations between PD-1, PD-L1 and CD8 expression (Table 3).

**Table 3:** Correlation between PD-1, PD-L1 expression and the presence of CD8^+^ **lymphocytes**

Subtype	PD-1_TIL_ and PD-L1	PD-1_TIL_ and CD8	PD-1_tumor_ and CD8	PD-L1 and CD8	PD-1^+^PD-L1^+^ and CD8
**OST**	-	-	-	-	-
**ES**	-	-	-	-	-
**ARMS**	-	-	-	-	-
**ERMS**	-	***p* = 0.006**^a^	-	*p* = 0.065^b^	-
**SyS**	-	-	-	-	-
**DSRCT**	-	-	-	-	-

Chi-square or Fisher’s exact analysis. -: not significant, *p*-value < 0.05 is considered significant, *p*-value < 0.1 is considered a trend, PD-1TIL: PD-1 expression on tumor infiltrating lymphocyte, PD-1_tumor_: PD-1 expression on tumor cell, OST: osteosarcoma, ES: Ewing sarcoma, ARMS: alveolar rhabdomyosarcoma, ERMS: embryonal rhabdomyosarcoma, SyS: synovial sarcoma, ^a^0% CD8^–^: PD-1TIL^+^ vs. 17% CD8^+^: PD-1TIL^+^, ^b^10% CD8^–^: PD-L1^+^ vs. 28% CD8^+^: PD-L1^+.^

### Correlation between PD-1, PD-L1, CD8^+^ expression and patients characteristics

Patient characteristics were specified per subtype and when available gender, age, tumor size, tumor location, tumor grade, metastases and treatment response were correlated to PD-1, PD-L1 and CD8^+^ expression (Tables 1, 4 and Supplementary Table 1). Male gender significantly correlated or had a trend with the presence of CD8^+^ lymphocytes in the tumor for Ewing sarcoma (*p =* 0.026) and DSRCTs (*p =* 0.088), respectively. In addition, soft tissue origin of Ewing sarcomas significantly correlated with PD-1 expression on the tumor (*p =* 0.007) (Table 4). In embryonal rhabdomyosarcoma small tumor size (< 5 cm) significantly correlated with CD8^+^ lymphocytes in the tumor (*p =* 0.022). In contrast, in osteosarcoma large tumor size showed a trend towards more CD8^+^ tumors (*p =* 0.085) (Table 4). In alveolar rhabdomyosarcoma, PD-L1^+^ and PD-L1^+^CD8^+^ tumors significantly correlated with low IRS grade (*p =* 0.005; *p =* 0.033), whereas PD-1^+^, PD-1^+^PD-L1^+^, PD-1^+^CD8^+^ and PD-1^+^PD-L1^+^CD8^+^ tumors showed a trend towards a low IRS grade (*p =* 0.071). PD-L1 expression also correlated with the absence of metastases in ARMS (*p =* 0.027) (Table 4). No correlation with age at diagnosis, presence of initial metastases or response to treatment existed in any of the subtypes (Table 4).

**Table 4:** Correlation between PD-1, PD-L1 expression and the presence of CD8+ lymphocytes and clinical-pathological characteristics per individual sarcoma subtype

Subtype	Characteristic	Expression						
		**PD-1**^+^	**PD-L1**^+^	**CD8**^+^	**PD-1**^+^**PD-L1**^+^	**PD-1**^+^**CD8**^+^	**PD-L1**^+^**CD8**^+^	**PD-1**^+^**PD-L1**^+^**CD8**^+^
**OST**	Gender	-	-	-	-	-	-	-
	Age	-	-	-	-	-	-	-
	Tumor size	-	-	*p* = 0.085^a^	-	-	-	-
	Initial metastases	-	-	-	-	-	-	-
	Occurrence metastases	-	-	-	-	-	-	-
**ES**	Gender	-	-	***p* = 0.026**^b^	-	-	-	-
	Age	-	-	-	-	-	-	-
	Tumor size	-	-	-	-	-	-	-
	Location (bone vs. soft tissue)	***p* = 0.007**^c^	-	-	-	*p* = 0.087^d^		
	Initial metastases	-	-	-	-	-	-	-
	Occurrence metastases	-	-	-	-	-	-	-
	Response to post-surgery treatment	-	-	-	-	-	-	-
**ARMS**	Gender	-	-	-	-	-	-	-
	Age	-	-	-	-	-	-	-
	Tumor size	-	-	-	-	-	-	-
	(un) favorable location	-	-	-	-	-	-	-
	IRS grade	*p* = 0.071^e^	***p* = 0.005**^f^	-	*p* = 0.071^g^	*p* = 0.071^h^	*p* = 0.033^i^	*p* = 0.071^j^
	Initial metastases	-	**-**	-	-	-	-	-
	Occurrence metastases	-	***p* = 0.027**^k^	-	-	-	-	-
	Response to post-surgery treatment	-	-	-	-	-	-	-
**ERMS**	Gender	-	-	-	-	-	-	-
	Age	-	-	-	-	-	-	-
	Tumor size	-	-	***p* = 0.022**^l^	-	-	-	-
	(un) favorable location	-	-	-	-	-	-	-
	IRS grade	-	-	-	-	-	-	-
	Initial metastases	-	-	-	-	-	-	-
	Occurrence metastases	-	-	-	-	-	-	-
	Response to post-surgery treatment	-	-	-	-	-	-	-
**SyS**	Gender	-	-	-	-	-	-	-
	Age	-	-	-	-	-	-	-
	Tumor size	-	-	-	-	-	-	-
	French grading	-	-	-	-	-	-	-
	Mitotic index	-	-	-	-	-	-	-
	Initial metastases	-	-	-	-	-	-	-
	Occurrence metastases	-	-	-	-	-	-	-
	Tumor necrosis	-	-	-	-	-	-	-
**DSRCT**	Gender	-	-	*p* = 0.088^m^	-	-	-	-
	Age	-	-	-	-	-	-	-
	Tumor size	-	-	-	-	-	-	-
	Initial metastases	-	-	-	-	-	-	-
	Occurrence metastases	-	-	-	-	-	-	-

OST: osteosarcoma, ES: Ewing sarcoma, ARMS: alveolar rhabdomyosarcoma, ERMS: embryonal rhabdomyosarcoma, SyS: synovial sarcoma, -: no significant correlation, *p*-value < 0.05 is considered significant, *p-*value < 0.1 is considered a trend.^a^92% CD8^+^: > 5 cm vs. 67% CD8^–^: > 5 cm, ^b^CD8^+^: 77% is male vs. CD8^–^: 37% is male, ^c^PD-1_tumor_: 50% soft tissue ES vs. 5% bone ES, ^d^PD-1_tumor_^+^CD8^+:^ 30% soft tissue ES vs. 5% bone ES, ^e^PD-1^+^: IRS1 vs. PD-1-: IRS2-4, ^f^PD-L1^+^: IRS1-2 vs. PD-L1^–^: IRS3-4, ^g^PD-1^+^PD-L1^+^: IRS1 vs. not PD-1^+^PD-L1^+^: IRS2-4, ^h^PD-1^+^CD8^+^: IRS1 vs. not PD-1^+^CD8^+^: IRS2-4, ^i^PD-L1^+^CD8^+^: IRS1/2 vs. not PD-L1^+^CD8^+^: IRS2-4, ^j^PD-1^+^PD-L1^+^CD8^+^: IRS1 vs. not PD-1^+^PD-L1^+^CD8^+^: IRS2-4, ^k^PD-L1^+^: no metastases vs. PD-L1^–^: 82% have metastases, ^l^74% CD8^–^: ≥ 5 cm vs. 36% CD8^+^: ≥ 5 cm, ^m^CD8^+^: 86% is male vs. CD8^–^: 25% is male.

### Association of clinical outcome with PD-1, PD-L1 and CD8 expression

**Figure 3:**
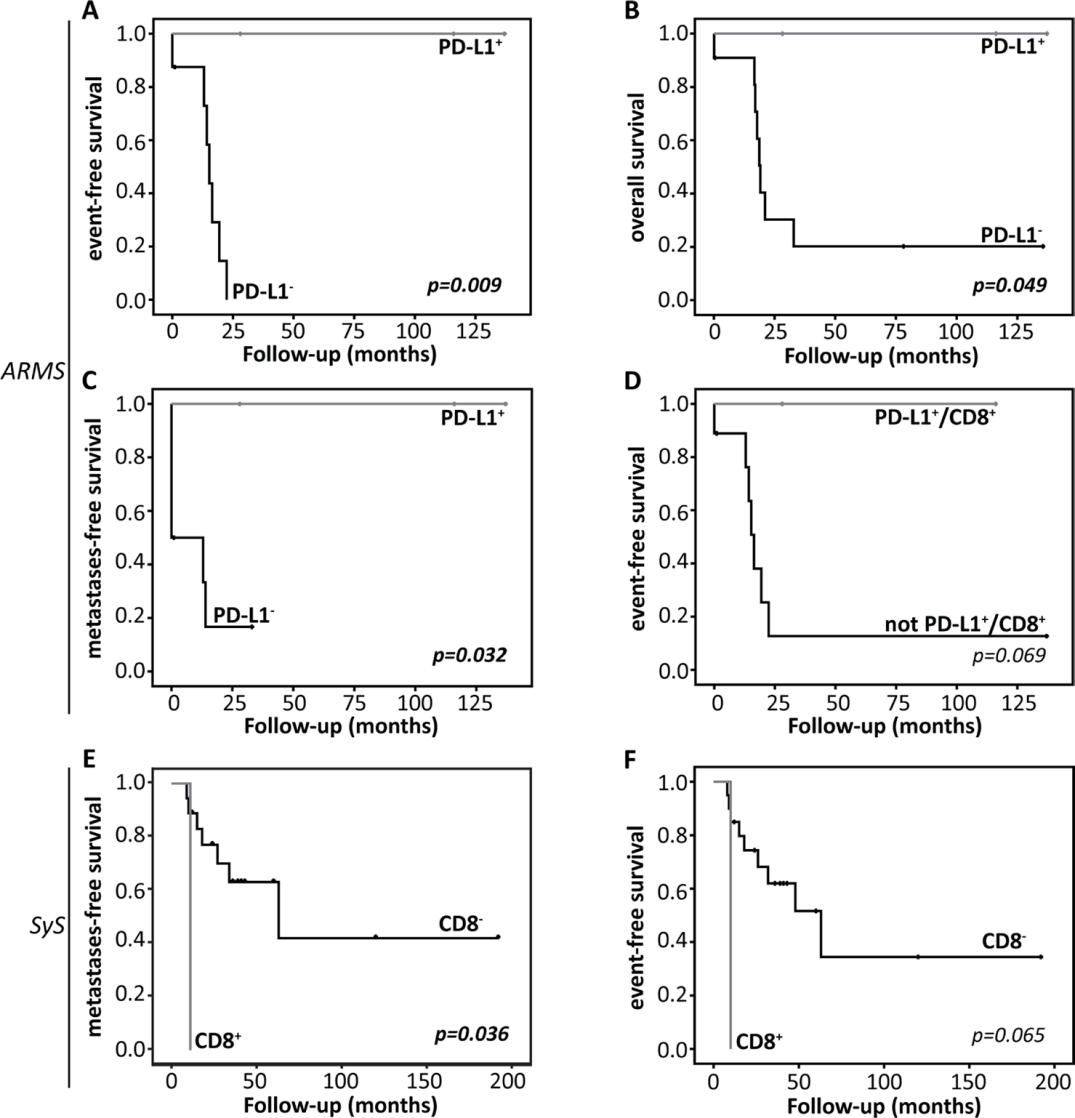

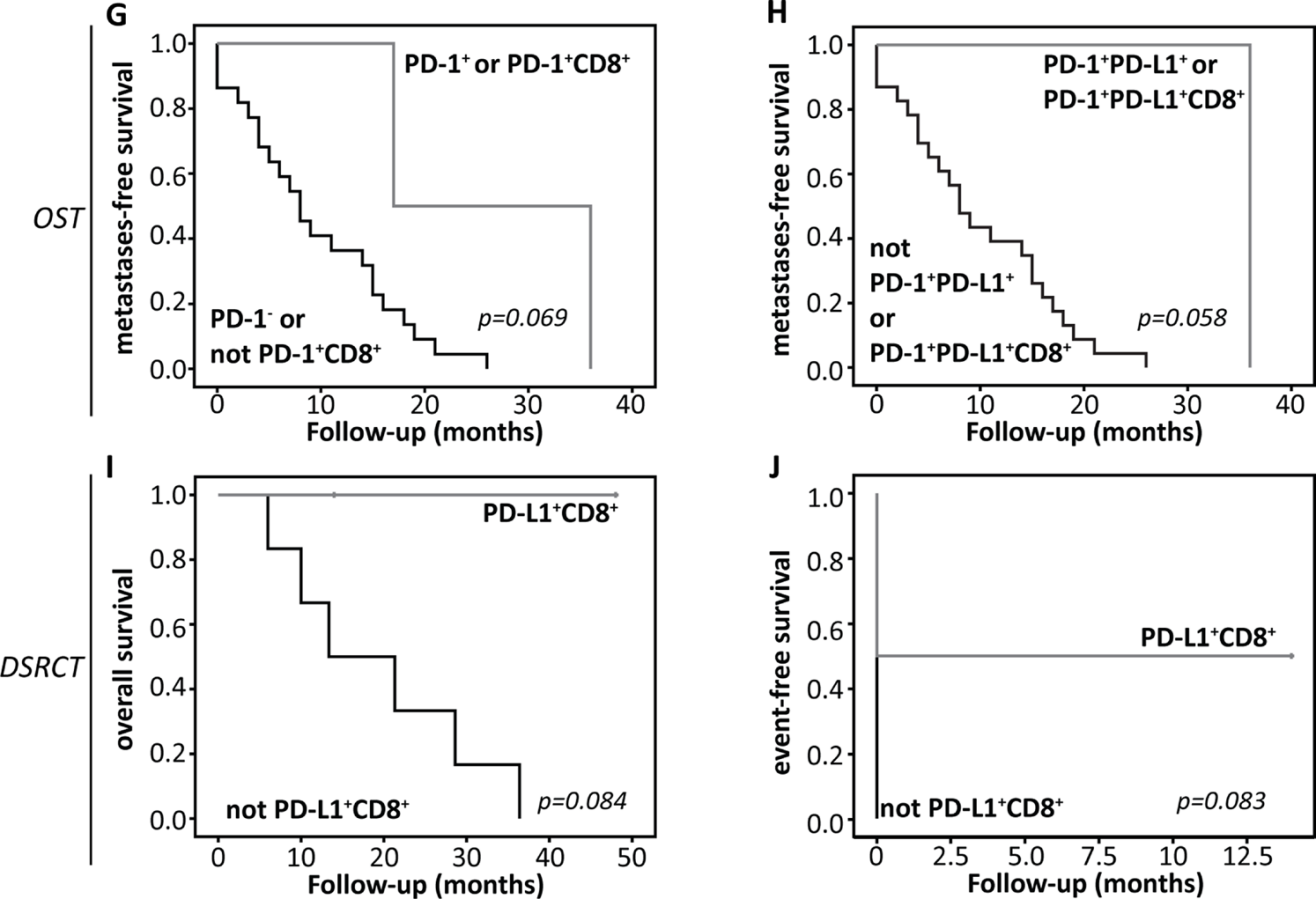
Kaplan-Meier survival analysis Overall survival (OS), event-free survival (EFS) or metastases-free survival (MFS) curves for (**A**–**D**) alveolar rhabdomyosarcoma (ARMS), (**E**–**F**) synovial sarcoma (SyS), (**G**–**H**) osteosarcoma (OST) or (**I**–**J**) desmoplastic small round cell tumors (DSRCT), according to PD-1, PD-L1 expression or presence of CD8^+^ lymphocytes in the tumor. Detailed information concerning statistical analysis is found in the Materials and Methods section.

PD-L1 expression correlated with better EFS, overall survival (OS) and metastases-free survival (MFS) in alveolar rhabdomyosarcoma (EFS: *p =* 0.009, OS: *p =* 0.049, MFS: *p =* 0.032) (Figure 3A–3C) and there was a trend towards better EFS for combined PD-L1 and CD8 expression (*p =* 0.069) (Figure 3D). In synovial sarcoma, the presence of high CD8^+^ in the tumor showed significantly worse MFS (*p =* 0.036) and a trend towards worse EFS (*p =* 0.065) (Figure 3E–3F). Whereas, expression of PD-1 (*p =* 0.069), PD-1^+^PD-L1^+^ (*p =* 0.058), PD-1^+^CD8^+^ (*p =* 0.069) and PD-1^+^PD-L1^+^CD8^+^ (*p =* 0.058) showed a trend towards better MFS in osteosarcoma (Figure 3G–3H). Better EFS was observed for the DSRCT patient with the PD-1^+^PD-L1^+^CD8^+^ tumor (*p =* 0.008) (data not shown). In addition, co-expression of PD-L1 and CD8 (*n =* 2) showed a trend towards better OS (*p =* 0.084) and EFS (event free during follow-up: *n =* 1, *p =* 0.083) (Figure 3I–3J).

### Assessment of PD-1 expression and response to nivolumab in **Ewing** sarcoma and DSRCT cell lines

**Figure 4:**
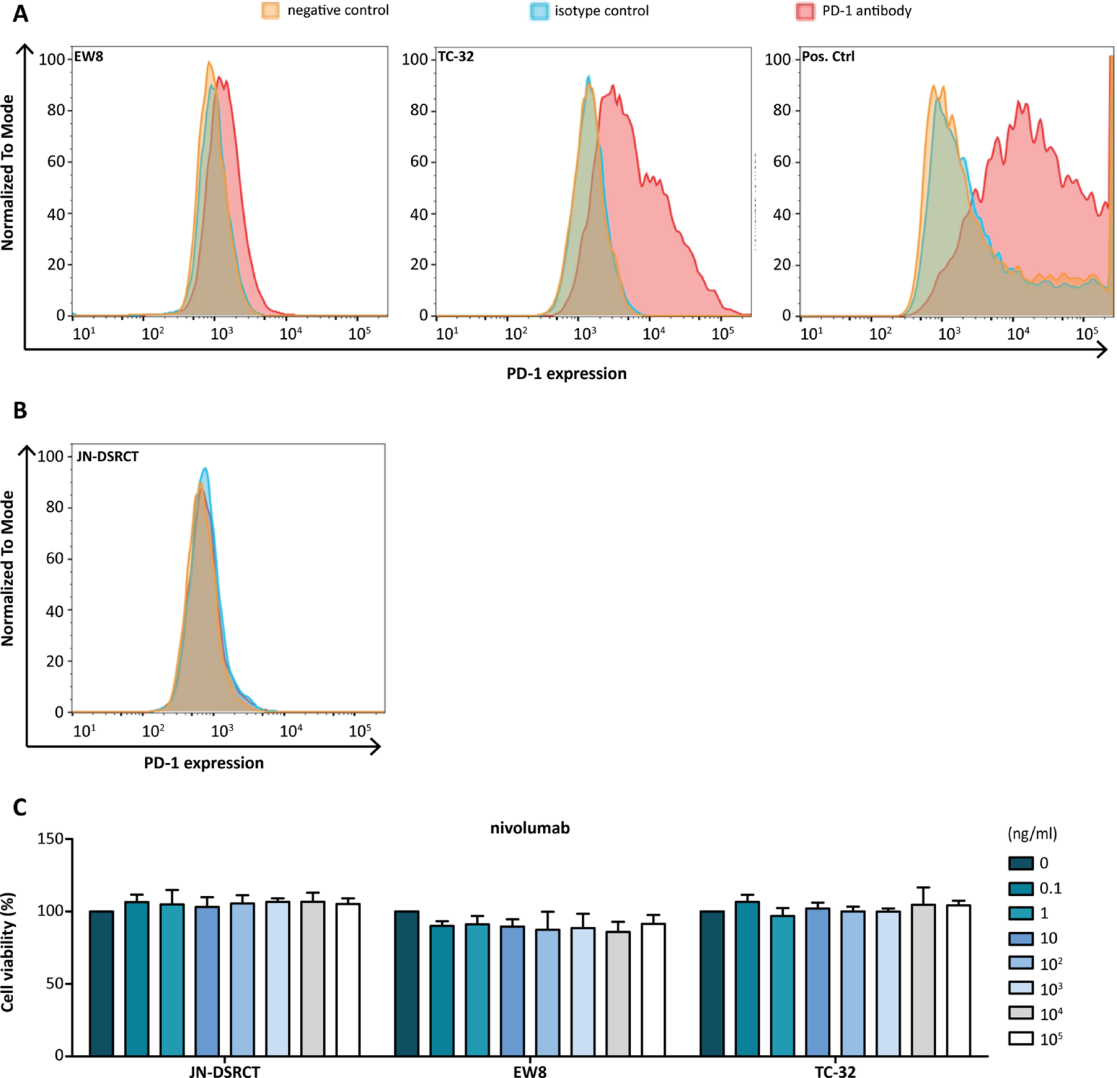
PD-1 expression and effect of anti-PD-1 antibody treatment in Ewing sarcoma cell lines (**A**) FACS analysis showed PD-1 expression on Ewing sarcoma cell line EW8 and TC-32. EW8 showed low PD-1 expression. PD-1-transfected CHO cells were used as a positive control. (**B**) The DSRCT cell line JN-DSRCT was negative for PD-1 and subsequently used as a negative control. (B) Anti-PD-1 antibody (nivolumab) treatment of JN-DSRCT, EW8, TC-32 cell lines had no effect on cell viability. Cell viability of untreated cells was set at 100%.

For functional analysis of PD-1 on Ewing sarcoma and DSRCT tumor cells, 10 Ewing sarcoma cell lines and 1 DSRCT cell line were assessed for PD-1 expression by FACS analysis. Ewing sarcoma cell lines EW8 and TC-32 showed PD-1 expression. EW8 showed lower levels of PD-1 (Figure 4A). All other cell lines, including the DSRCT cell line, were negative for PD-1 (Figure 4B, Supplementary Figure 1). *In vitro* treatment of EW8 and TC-32 with anti-PD-1 antibody nivolumab did not affect cell viability (Figure 4C).

## DISCUSSION

Children and AYA suffering from sarcoma are in need of novel therapies, as the current poor survival rates and the treatment-associated toxicities have a major impact on life expectancy and quality of life [18, 19]. In the current study we explored the potential of PD-1 blockade in sarcoma subtypes and showed that expression and clinical association of PD-1 and PD-L1 and the presence of CD8^+^ lymphocytes is sarcoma subtype dependent. In addition, for the first time PD-1 expression was observed on Ewing sarcoma and DSRCT tumor cells.

Osteosarcoma showed low levels of PD-1 and PD-L1 expression and higher levels of CD8^+^ lymphocytes in the tumor (Table 2). Recently, PD-L1 expression was shown to be associated with worse 5-year EFS and higher infiltration of immune cells [20, 21]. In addition, PD-L1 expression was shown to be higher when whole slide sections were assessed compared to tissue micro array (TMA) [21]. In the current study, 46 primary tumors were examined on TMA and when considering the previously found discrepancy between TMA and whole slide analysis, the low PD-1 and PD-L1 expression levels could be the result of an underestimation of the actual expression in osteosarcoma. Another explanation could be that in particular osteosarcoma metastases express PD-1 and PD-L1 [20, 22]. Examination of metastatic samples was beyond the scope of this study and we could thus not verify this finding. High levels of CD8^+^ lymphocytes in the tumor are in line with previous findings, as several studies showed PD-1^+^ immune cells in both primary and metastatic osteosarcoma patients [20–23]. In the current study, immune checkpoint expression showed a positive correlation with MFS (Figure 3G–3H). A possible explanation could be that PD-1 and CD8 expression suggest higher anti-tumor immune pressure, resulting in a response of the tumor cell by increasing PD-L1 on the cell membrane. Survival most likely depends on the balance between anti-tumor immunity and tumor evasion. Until this balance switches from anti-tumor immunity towards tumor evasion via PD-1 and PD-L1 interaction, better control of tumor progression could lead to prolonged metastases-free survival [24–26].

Interestingly, a subset of Ewing sarcoma showed PD-1 expression on the tumor cells, instead of on the TILs (Table 2, Figure 2B). In melanoma and esophageal adenocarcinoma PD-1 expression was similarly seen on tumor cells. In melanoma targeting of tumor-intrinsic PD-1 *in vivo* was found to reduce tumor growth and in esophageal adenocarcinoma PD-1 expression on tumor cells associated with advanced disease [27–29]. In addition, efficacy of PD-1 blockade was reported in one Ewing sarcoma patient with a sustained response to treatment [30]. Our functional analysis of PD-1 on ES tumor cells showed PD-1 expression on 2/10 ES cell lines, however; targeting of PD-1 *in vitro* did not have an effect on cell viability. Of note, little is known about the mechanism of action of PD-1 on a tumor cell and it could be that, similar to what was found in melanoma, interaction with tumor cell intrinsic PD-L1 leads to tumor progression [28]. *In vivo* studies will help to determine the potential of PD-1 targeting in Ewing sarcoma. Others showed the expression of PD-1 on TILs and PD-L1 in Ewing sarcoma. Previous studies showed a higher percentage of positive tumors, although sample sizes were significantly lower compared to our cohort [13–15, 31]. Similar to a previous finding [13], bone and soft-tissue Ewing sarcoma showed a difference in expression. In order to determine whether tissue of origin might influence the potential of PD-1 blockade, further research is necessary to verify the differences between bone and soft tissue Ewing sarcoma. Our findings suggest that a subset of ES patients might benefit from PD-1 blockade, although further research is necessary.

PD-1^+^ TILs and PD-L1 have been associated with poor prognosis in STS [14, 31]. In comparison to previous findings, the current study showed lower levels of PD-L1 expression for both alveolar and embryonal rhabdomyosarcoma [14, 15, 31]. However, this might be explained by the differences in sample size and antibodies used. In addition, we only examined fusion-positive ARMS which might have an influence on the expression level. PD-L1 expression in alveolar rhabdomyosarcoma associated with better EFS, OS and MFS (Figure 3A, 3C). In addition, PD-L1 alone or co-expression with CD8 significantly correlated with a lower IRS grade and PD-L1 expression alone correlated with the absence of metastases (Table 4). Similar to other tumor types [32], the positive correlation of PD-L1 could, instead of tumor immune evasion, indicate a response of the tumor cell to high immune pressure by CD8^+^ lymphocytes and could lead to a better prognosis as long as the balance is in favor of anti-tumor immunity [24–26].

For embryonal rhabdomyosarcoma no association with survival was observed. However, PD-1 expression correlated with the presence of CD8^+^ lymphocytes in the tumor, and a trend for co-expression of PD-L1 and CD8 was seen (Table 3). High PD-L1 expression and high numbers of CD8^+^ lymphocytes were present in a small percentage of tumors (Table 2). The correlation with PD-1 and the presence of high PD-L1 and CD8^+^ in a small percentage of tumors suggest that PD-1 blockade might be a therapeutic option for a subset of embryonal rhabdomyosarcoma patients.

Synovial sarcoma showed low levels of PD-1 and PD-L1 expression and relatively high CD8 expression (Table 2). The presence of high levels of CD8^+^ lymphocytes in the tumor correlated with a worse MFS and showed a trend towards worse EFS (Figure 3E–3F). Recently, Nowicki *et al.* also examined PD-1, PD-L1 and CD8 in synovial sarcomas [33]. They showed a similar association for CD8 with worse progression-free survival (PFS), in addition to worse PFS for PD-1 expression. The expression of PD-1 and CD8 on lymphocytes in the tumor are in line with our findings. However, the expression of PD-L1 on synovial sarcoma cells could not be observed in our cohort. Overall, expression of immune checkpoint could be a negative prognostic marker in synovial sarcoma and further research into the expression of PD-1, PD-L1 and CD8 is of interest.

For the first time PD-1 and CD8 expression are described in a cohort of DSRCTs. Two studies have previously examined PD-L1 expression in DSRCTs. Similar to our finding, Movva *et al.* showed PD-L1 expression, although in a small cohort of DSRCTs (*n =* 2) [16]. Inaguma *et al.* examined a larger cohort and did not detect PD-L1 [17]. Despite the differences in expression, in our study the majority of DSRCTs showed high PD-1 expression on tumor cells and the co-expression of PD-1, PD-L1 and CD8 suggests that the PD-1/PD-L1 axis could be present in a small subset of DSRCTs (Table 2) (Figure 2A). Interestingly, similar to Ewing sarcoma, DSRCT has a characteristic translocation involving *EWSR1*, calling for investigation into the effects of this gene on PD-1 expression. Better EFS was observed for the PD-1^+^PD-L1^+^CD8^+^ patient, This patient showed no events during the follow-up period (14 months) and had continued survival at the time of data acquisition. Similar to other subtypes, this suggests a more dominant anti-tumor immune response in this patient. Since only a small cohort has been examined more research is necessary to strengthen the detected expression levels and possible clinical implications of immune checkpoint expression in DSRCTs.

Overall, the current study shows both similarities and differences with previously found expression data. The differences in expression could however be the result of different antibodies for immunohistochemistry, as sensitivity between antibodies differs. In addition, as no consensus has been reached for expression analysis, the threshold for positive expression varies between studies (Supplementary Table 2) [32]. Moreover, recent studies have shown that expression levels, on their own, might not be sufficient to predict a response to immune checkpoint blockade. Factors that could be of importance are the level of PD-1^+^ TILs following treatment [34], PD-L1 on TILs before treatment [35] and the mutational load of the tumor [36]. Further research is needed to determine whether these predictive markers also apply to sarcomas. In addition, we only assessed PD-L1 expression, PD-L2 is a second ligand of PD-1 and similarly capable of reducing T cell activity [37]. Even though PD-L1 has been shown to have a higher frequency in malignancies, the analysis of PD-L2 expression in sarcomas could be of interest [38].

In conclusion, expression and clinical associations of PD-1, PD-L1 and CD8^+^ lymphocytes in the tumor are sarcoma subtype dependent. This difference between subtypes underlines the necessity for further research into the specific mechanistic aspects of immune evasion per subtype in order to determine whether PD-1 blockade is of therapeutic interest. In addition, for the first time, PD-1 expression was found to be present on Ewing sarcoma and DSRCT tumor cells instead of TILs. Given the tremendous need for new therapeutic approaches, in particular for DSRCTs, these observations will be further explored.

## MATERIALS AND METHODS

### Patient characteristics

Primary tumors of 208 sarcoma patients were examined. Table 1 shows the number of tumors per subtype, gender, age at diagnosis, occurrence of (initial) metastases, available follow-up data and, when applicable, genetic translocation for each individual subtype. Supplementary Table 1 shows tumor size, tissue of origin, treatment response and, when applicable, tumor location ((un)favorable), tumor grade and the occurrence of an event or death of disease (DOD).

The study was performed in accordance with the Code of Conduct of the Federation of Medical Scientific Societies in the Netherlands.

### Immunohistochemistry

Immunohistochemistry analysis was performed to specifically investigate PD-1 and PD-L1 expression and the presence of CD8^+^ lymphocytes in the tumor. Tonsil (PD-L1^+^, PD-1^+^ and CD8^+^) and appendix (CD8^+^ and PD-1^+^) served as positive controls. Immunohistochemistry was performed on 4µm-thick formalin-fixed paraffin-embedded (FFPE) sections of DSRCT and tissue microarrays (TMAs) (core size 1 or 2 mm) of osteosarcoma, Ewing sarcoma, alveolar rhabdomyosarcoma, embryonal rhabdomyosarcoma and synovial sarcoma to allow simultaneous examination of patient specimens under identical conditions. Sections were deparaffinized in xylol and rehydrated through a graded ethanol into water series. Antigen retrieval was performed by heating the slides in 10 mM sodium citrate buffer, pH6 (PD-1 and PD-L1) or in EDTA buffer, pH9 (CD8) for 10 min at 100°C. Endogenous peroxidase activity was blocked with 3% H_2_O_2_ in methanol or distilled water for 10 min at RT. Subsequently, sections were incubated with monoclonal rabbit anti-PD-L1 (1:200, clone E1L3N, Cell Signaling Technology), monoclonal mouse anti-PD-1 (1:20, clone MRQ-22, Cell Marque) or monoclonal mouse anti-CD8 (1:80, clone C8/144B, Dako) in antibody diluent in a humidified chamber overnight at 4°C (PD-L1) or 1h at RT (PD-1 and CD8). For PD-1 a 15min incubation step at RT with post-antibody blocking (BrightVision Ultimate Plus, ImmunoLogic) was performed. Next, sections were incubated with Poly-HRP-Anti-mouse/rabbit/rat IgG (ImmunoLogic) in PBS-Tween20 0.05% (1:1) for 30 min at RT. Antibody binding was visualized using bright 3,3′-diaminobenzidine (DAB) (ImmunoLogic) for 7 min at RT. Finally, slides were counterstained with haematoxylin, dehydrated and coverslipped.

PD-1 expression on tumor infiltrating cells was scored as either negative (–) or positive (+). PD-1 and PD-L1 expression on the tumor cell was scored as < 10% (negative), 10–50% or ≥ 50% positive tumor cells. All CD8-positive (CD8^+^) cells were counted and subdivided in three categories: < 10 (–), 10–50 or ≥ 50 positive cells in the tumor. Expression in ≥ 50% of the tumor cells and > 50 CD8^+^ cells in the tumor were considered high expression (PD-1_high_, PD-L1_high_, CD8_high_). Digital images were generated with VisionTek™ (Sakura, version 2.6) and analyzed at 20x magnification. An example of each staining is given in Figure 2.

### Statistical analysis

Statistical analyses were performed using IBM SPSS Statistics 22 and *p-value* < 0.05 was considered significant and *p-value* < 0.1 was considered a trend. Relations between categorical parameters were assessed by Chi-square or Fisher’s exact testing and associations with overall (OS), event-free (EFS) and metastases-free survival (MFS) were assessed by the Kaplan-Meier method with Log rank test. Tumors positive for one marker or a combination were compared to tumors negative for that particular marker or combination. A distinction between the different levels of expression (< 10, 10–50, ≥ 50) was made in the analysis.

### Cell lines

Ewing sarcoma cell lines ES1, ES2, ES4, ES7, ES8 and EW8 were generously provided by dr. Peter Houghton of the Pediatric Preclinical Testing Program (PPTP). Ewing sarcoma cell lines TC-32, SK-N-DW, SK-N-MC and CHP-100 were generously provided by dr. Friederike Meyer-Wentrup of the Princess Máxima center for Pediatric Oncology and the DSRCT cell line was generously provided by dr. Janet Shipley of The Institute of Cancer Research. All cell lines were authenticated by determining the disease-specific genetic translocation (EWSR1-FLI1/ERG for Ewing sarcoma and EWSR1-WT1 for DSRCT). Ewing sarcoma cell lines were cultured in RPMI-1640 medium (ES1, ES2, ES4, ES7, ES8 and EW8, Lonza Benelux BV, Breda, The Netherlands) or DMEM medium (TC-32, SK-N-DW, SK-N-MC and TC-32CHP-100, Lonza). JN-DSRCT was cultured in DMEM/F12 GlutaMAX™ medium (Gibco). *PDCD1-*transfected CHO cell line was culture in Ham’s F12 medium (Gibco). Media were supplemented with 10% fetal bovine serum (FBS) (Gibco) and 1% penicillin/streptomycin (Lonza). All cell lines were cultured in a humidified atmosphere of 5% CO_2_/95% O_2_ air at 37°C.

### Flow cytometry

PD-1 expression was analyzed by flow cytometry: 1 × 10^5^ cells per cell line were washed in PBS with 2% FBS (Gibco) and 0.5% BSA (Sigma) (PBA) followed by 20 min incubation at RT with either mouse anti-human PD-1 (CD279) antibody (BB515, 1:20 in PBA, BD Horizon) or mouse IgG1 κ isotype antibody (BB515 Clone X40, 1:80 in PBA, BD Horizon). CHO cell line transfected with a pcDNA3.1^+^/C-(K)DYK vector enriched with *PDCD1* for 48h acted as a positive control (Lipofectamine 3000 Transfection kit™, Thermo Scientific). Cells were washed and dissolved in PBA followed by flow cytometry using FACS Verse (BD Biosciences). Data analysis was performed using FLowJo version 10.0.7.

### Cell viability assay

Treatment effects of nivolumab (Opdivo^®^ 10 mg/ml) were assessed by MTT assay. All cell lines were seeded in quadruplicate into flat-bottomed 96-well plates at 5000 cells per well and allowed to adhere. Increasing concentrations of nivolumab (0–0.1 mg/ml) were added followed by 72 h (EW8, ES1) or 120 h (TC-32) incubation at 37°C, based on estimated growth rate. Subsequently, 20 μl MTT (5 mg/ml in PBS) was added and cells were again incubated for 3.5 h at 37°C. Medium was removed and the formed formazan crystals were dissolved in 150 μl acidified isopropanol solution. Absorbance was read at 560 nm using an ELISA reader. Data analysis was performed with GraphPad Prism (Version 5.03).

## SUPPLEMENTARY MATERIALS FIGURE AND TABLES





## Abbreviations

DSRCT: desmoplastic small round cell tumor
EFS: event-free survival
MFS: metastases-free survival
NSCLC: non-small cell lung cancer
OS: overall survival
PD-1: programmed cell death-1
PD-L1: programmed death ligand-1
STS: soft tissue sarcoma
TIL: tumor infiltrating lymphocyte
TL: T lymphocytes
TMA: tissue microarray
TME: tumor microenvironment
