# Supplementary material for: Expression and clinical association of programmed cell death-1, programmed death-ligand-1 and CD8+ lymphocytes in primary sarcomas is subtype dependent
Source: Oncotarget. 2017 Jul 7;8(41):71371–84. doi: 10.18632/oncotarget.19071 (PMC5642642; doi:10.18632/oncotarget.19071)
Supplement: Supplementary file 1 [file oncotarget-08-71371-s001.pdf]

Expression and clinical association of programmed cell death-1, programmed death-ligand-1 and CD8^{+} lymphocytes in primary sarcomas is subtype dependent

SUPPLEMENTARY MATERIALS

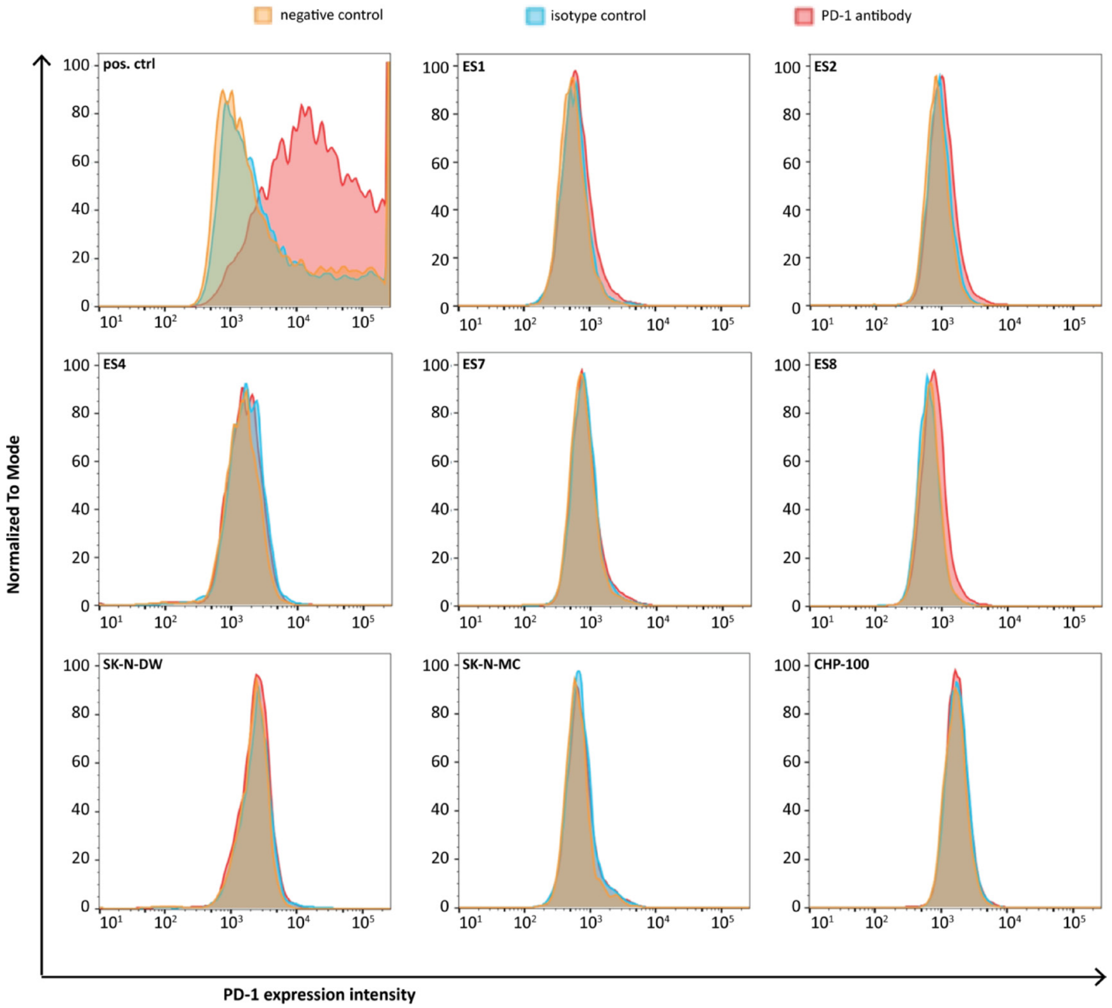

Supplementary Figure 1: PD-1 expression in Ewing sarcoma and DSRCT cell lines. FACS analysis showed no expression of PD-1 on Ewing sarcoma cell lines ES1, ES2, ES4, ES7, ES8, SK-N-DW, SK-N-MC and CHP-100. PD-1-transfected CHO cells were used as positive control

Supplementary Table 1: Patient characteristics per individual sarcoma subtype - continued. See Supplementary_Table_1

Supplementary Table 2: Number of tumors included, antibodies used and expression analysis for the representative studies

**Number of tumors**						
Subtype	*Paydas et al. [1]*	*Kim et al. [2]*	*Chowdhury et al. [3]*	*Movva et al. [4].*	*Inaguma et al. [5]*	*Current study*
**OST**	*n* = 10	*n* = 0	*n* = 15	*n* = 7	*n* = 0	*n* = 46
**ES**	*n* = 3	*n* = 18	*n* = 14	*n* = 7	*n* = 38	*n* = 32
**ARMS**	*n* = 0	*n* = 32[a]	*n* = 14	*n* = 8[a]	*n* = 42	*n* = 20
**ERMS**	*n* = 0		*n* = 16		*n* = 54	*n* = 77
**SyS**	*n* = 4	*n* = 19	*n* = 0	*n* = 4	*n* = 34	*n* = 22
**DSRCT**	*n* = 0	*n* = 0	*n* = 0	*n* = 1 (PD-L1 only)	*n* = 14 (PD-L1 only)	*n* = 11
**Antibody**						
**PD-1**	MRQ-22 (Cell Marque)	n.a.	PAB19608 (Abnova)	BD Pharmingenb	n.a.	MRQ-22 (Cell Marque)
**PD-L1**	AM26531AF-N (Acris)	130021 (R&D)	Ab58810 (Abcam)	R&D[b]	E1L3N (Cell Signaling)	E1L3N (Cell Signaling)
**CD8**	n.a.	n.a.	MAB11248 (Abnova)	n.a.	n.a.	C8/144b (Dako)
**Expression analysis**						
**PD-1**	–, +, ++, +++	n.a.	#PD1$^+$CD8$^+$ TILs	≥ 1+ (TIL/HPF)	n.a.	Negative/positive lymphocytes or < 10%, 10–50%, ≥ 50% pos.
**PD-L1**	–, +, ++, +++	–, +/++, +++ and 0, < 10%, 10–50%, 50% pos.	> 5% pos.	≥ 2+ and ≥ 5% pos.	> 5% pos.	< 10%, 10–50%, ≥ 50% pos.
**CD8**	n.a.	n.a.	> 20 (high)	n.a.	n.a.	< 10, 10–50, ≥ 50 pos.

OST: osteosarcoma, ES: Ewing sarcoma, ARMS: alveolar rhabdomyosarcoma, ERMS: embryonal rhabdomyosarcoma, SyS: synovial sarcoma,DSRCT: desmoplastic small round cell tumor, [a]no further specification of rhabdomyosarcoma, PD-1: Programmed cell death-1, PD-L1: programmed death ligand 1, n.a.: not applicable, [b]no further specification of antibody, –: no expression, +: weak expression, ++: moderate expression, +++: strong expression, pos.: positive tumor cells.

REFERENCES

1. Paydas S, Bagir EK, Deveci MA, Gonlusen G. Clinical and prognostic significance of PD-1 and PD-L1 expression in sarcomas. Medical oncology (Northwood, London, England). 2016; 33:93.
2. Kim C, Kim EK, Jung H, Chon HJ, Han JW, Shin KH, Hu H, Kim KS, Choi YD, Kim S, Lee YH, Suh JS, Ahn JB, et al. Prognostic implications of PD-L1 expression in patients with soft tissue sarcoma. BMC Cancer. 2016; 16:434.
3. Chowdhury F, Dunn S, Mitchell S, Mellows T, Ashton-Key M, Gray JC. PD-L1 and CD8+PD1+ lymphocytes exist as targets in the pediatric tumor microenvironment for immunomodulatory therapy. Oncoimmunology. 2015; 4:e1029701.
4. Movva S, Wen W, Chen W, Millis SZ, Gatalica Z, Reddy S, von Mehren M , Van Tine BA. Multi-platform profiling of over 2000 sarcomas: identification of biomarkers and novel therapeutic targets. Oncotarget. 2015; 6:12234–12247. https://doi.org/10.18632/oncotarget.3498.
5. Inaguma S, Wang Z, Lasota J, Sarlomo-Rikala M, McCue PA, Ikeda H, Miettinen M. Comprehensive Immunohistochemical Study of Programmed Cell Death Ligand 1 (PD-L1): Analysis in 5536 Cases Revealed Consistent Expression in Trophoblastic Tumors. The American journal of surgical pathology. 2016; 40:1133–1142.
