# Supplementary material for: Expression and clinical association of programmed cell death-1, programmed death-ligand-1 and CD8+ lymphocytes in primary sarcomas is subtype dependent
Source: Oncotarget. 2017 Jul 7;8(41):71371–84. doi: 10.18632/oncotarget.19071 (PMC5642642; doi:10.18632/oncotarget.19071)
Supplement: Supplementary file 2 [file oncotarget-08-71371-s002.docx]

Table S1 **Patient characteristics per individual sarcoma subtype - continued**

| *Tumor type* | *Characteristics* |  | *N (%)* |
| --- | --- | --- | --- |
| **OST (N=46)** | Tumor size | <5cm | 7(15) |
|  |  | 5-10cm | 21 (46) |
|  |  | >10cm | 11 (24) |
|  |  | Unknown | 7 (15) |
|  | Tissue of origin | Bone | 46 (100) |
|  | Treatment response | <90% necrosis | 17 (37) |
|  |  | ≥90% necrosis | 6 (13) |
|  |  | Unknown | 23 (50) |
| **ES (N=32)** | Tumor size | <5cm  5-10cm  >10cm  Unknown | 4 (12.5)  14 (44)  10 (31)  4 (12.5) |
|  | Tissue of origin | Bone | 21 (66) |
|  |  | Soft tissue | 10 (31) |
|  |  | Unknown | 1 (3) |
|  | Event/DOD | Event | 11 (34) |
|  |  | DOD | 12 (38) |
|  | Treatment response | <90% necrosis | 7 (22) |
|  |  | ≥90% necrosis  Unknown | 10 (31)  15 (47) |
| **ARMS (N=20)** | Tumor size | <5cm | 3 (15) |
|  |  | ≥5cm | 2 (10) |
|  |  | Unknown | 15 (75) |
|  | Tissue of origin | Parameningeal | 4 (20) |
|  |  | Perineum/peri-anal | 3 (15) |
|  |  | Orbit | 1 (5) |
|  |  | Extremities | 6 (30) |
|  |  | Urogenital track | 2 (10) |
|  |  | Head and neck | 2 (10) |
|  |  | Abdomen | 1 (5) |
|  |  | Unknown | 1 (5) |
|  | Location tumor | Favorable | 12 (60) |
|  |  | Unfavorable | 7 (35) |
|  |  | Unknown | 1 (5) |
|  | IRS grade | Grade 1 | 1 (5) |
|  |  | Grade 2 | 2 (10) |
|  |  | Grade 3 | 3 (15) |
|  |  | Grade 4 | 8 (40) |
|  |  | Unknown | 6 (30) |
|  | Event/DOD (n=14) | Event | 10 (50) |
|  |  | DOD | 8 (40) |
|  | Treatment response | CR | 1 (5) |
|  |  | PD | 3 (15) |
|  |  | Unknown | 16 (80) |
| **ERMS (N=77)** | Tumor size | <5cm | 15 (19) |
|  |  | ≥5cm | 22 (29) |
|  |  | Unknown | 40 (52) |
|  | Tissue of origin | Urogenital | 32 (41) |
|  |  | Parameningeal | 10 (13) |
|  |  | Orbita | 9 (12) |
|  |  | Prostate/bladder | 7 (9) |
|  |  | Head and neck | 5 (6) |
|  |  | Extremities | 3 (4) |
|  |  | Retroperitoneal | 3 (4) |
|  |  | Hip | 2 (3) |
|  |  | Trunk | 2 (3) |
|  |  | Other | 3 (4) |
|  |  | Unknown | 1 (1) |
|  | Location of tumor | Favorable | 28 (37) |
|  |  | Unfavorable  Unknown | 48 (62)  1 (1) |
|  | IRS grade | Grade 1 | 27 (35) |
|  |  | Grade 2 | 3 (4) |
|  |  | Grade 3 | 14 (18) |
|  |  | Grade 4 | 4 (5) |
|  |  | Unknown | 29 (38) |
|  | Treatment response | Vital and differentiation | 13 (17) |
|  |  | Complete response | 5 (6) |
|  |  | unknown | 59 (77) |
| **SyS (N=22)** | Tumor size | <5 cm | 6 (27) |
|  |  | 5-10cm | 9 (41) |
|  |  | >10cm | 1 (5) |
|  |  | Unknown | 6 (27) |
|  | Tissue of origin | Extremities | 15 (68) |
|  |  | Head and neck | 3 (13.5) |
|  |  | Other | 3 (13.5) |
|  |  | Unknown | 1 (5) |
|  | French grading | Grade 2 | 18 (82) |
|  |  | Grade 3 | 2 (9) |
|  |  | Unknown | 2 (9) |
|  | Mitotic index (per 10 HPF) | 0-9 | 15 (68) |
|  |  | 10-19 | 4 (18) |
|  |  | >19 | 1 (5) |
|  |  | Unknown | 2 (9) |
|  | Tumor necrosis | No necrosis | 17 (77) |
|  |  | <50% | 2 (9) |
|  |  | ≥50% | 1 (5) |
|  |  | Unknown | 2 (9) |
| **DSRCT (N=11)** | Tumor size | <5 cm | 1 (9) |
|  |  | ≥5 cm | 8 (73) |
|  |  | Unknown | 2 (18) |
|  | Tissue of origin | Abdomen | 9 (82) |
|  |  | Kidney | 2 (18) |
| *N: number of primary tumors, OST: osteosarcoma, ES: Ewing sarcoma, ARMS: alveolar rhabdomyosarcoma, ERMS: embryonal rhabdomyosarcoma, SyS: synovial sarcoma, DSRCT: desmoplastic small round cell tumor, DOD: death of disease, OS: overall survival, EFS: event-free survival, CR: complete response, PD: progressive disease,  ^a^percentage calculation: (total initial metastases/total with metastases)*100%* | | | |
